# Supplementary material for: A complete hierarchy for the pure state marginal problem in quantum mechanics
Source: Nat Commun. 2021 Feb 12;12:1012. doi: 10.1038/s41467-020-20799-5 (PMC7881147; doi:10.1038/s41467-020-20799-5)
Supplement: Supplementary file 1 — Supplementary Information [file 41467_2020_20799_MOESM1_ESM.pdf]

Supplementary Information for "A complete hierarchy for the pure state marginal problem in quantum mechanics"

Xiao-Dong Yu, Timo Simnacher, Nikolai Wyderka, H. Chau Nguyen, and Otfried Gühne

Supplementary Note 1: Existence and uniqueness of the symmetrized Φ_{AB} for AME states

Before proving the existence and uniqueness of the symmetrized Φ_{AB}, we show how to simplify the constraints in Equations (27, 28) by taking advantage of Equation (35). The meaning of this simplification is two-fold: first, it gives an intuition about why the symmetrized Φ_{AB} is uniquely determined; second, it can be directly generalized to other marginal problems, such as the m-uniform states and quantum codes, in which the symmetrized Φ_{AB} are no longer uniquely determined. Recall the symmetrized Φ_{AB} is of the form

$$\Phi_{AB} = \sum_{i=0}^{n} x_i \mathcal{P}\{V^{\otimes i} \otimes \mathbb{1}^{\otimes(n-i)}\}, \tag{1}$$

then the constraints in Equations (27, 28) can be simplified as follows:

• **Normalization constraint $\mathrm{Tr}(\Phi_{AB}) = 1$:**

$$\mathrm{Tr}(\Phi_{AB}) = \mathrm{Tr}\left[\sum_{i=0}^{n} x_i \mathcal{P}\{V^{\otimes i} \otimes \mathbb{1}^{\otimes(n-i)}\}\right] = \sum_{i=0}^{n} \binom{n}{i} d^{2n-i} x_i = 1. \tag{2}$$

• **Symmetric subspace constraint $V_{AB}\Phi_{AB} = \Phi_{AB}$:**

$$V_{AB}\Phi_{AB} = V^{\otimes n}\Phi_{AB} = \sum_{i=0}^{n} x_i \mathcal{P}\{V^{\otimes(n-i)} \otimes \mathbb{1}^{\otimes i}\} = \sum_{i=0}^{n} x_i \mathcal{P}\{V^{\otimes i} \otimes \mathbb{1}^{\otimes(n-i)}\}, \tag{3}$$

which implies that

$$x_i = x_{n-i} \quad \forall\, i = 0, 1, \ldots, n-r-1, \tag{4}$$

where $r = \lfloor n/2 \rfloor$.

• **Marginal constraints $\mathrm{Tr}_{A_{I^c}}(\Phi_{AB}) = \frac{\mathbb{1}_{d^r}}{d^r} \otimes \mathrm{Tr}_A(\Phi_{AB})$:**

Because Φ_{AB} is invariant under permutations $\Pi \in S_n$, it is sufficient to consider $I^c = \{1, 2, \ldots, n-r\}$. Further, as $\frac{\mathbb{1}_{d^r}}{d^r} \otimes \mathrm{Tr}_A(\Phi_{AB}) \propto \mathbb{1}_{d^{n+r}}$, it must also hold that $\mathrm{Tr}_{A_{I^c}}(\Phi_{AB}) \propto \mathbb{1}_{d^{n+r}}$. Hence, all terms that contain V in $\mathrm{Tr}_{A_{I^c}}(\Phi_{AB})$ must be zero. Thus, the marginal constraints $\mathrm{Tr}_{A_{I^c}}(\Phi_{AB}) = \frac{\mathbb{1}_{d^r}}{d^r} \otimes \mathrm{Tr}_A(\Phi_{AB})$ are equivalent to

$$\sum_{i=0}^{n-r} \binom{n-r}{i} d^{n-r-i} x_{s+i} = 0 \quad \forall\, s = 1, 2, \ldots, r. \tag{5}$$

Supplementary Equations (2, 4, 5) provide $n+1$ linear equations, which can uniquely determine the $n+1$ parameters $(x_0, x_1, \ldots, x_n)$ in Φ_{AB}.

To rigorously prove the existence and uniqueness of Φ_{AB} constrained by Supplementary Equations (2, 4, 5), we take advantage of the following lemma; for more details about the dual basis, see e.g., Ref. [1].

Lemma 1. *Let $\{|x_i\rangle\}_i$ be a basis for a finite-dimensional Hilbert space, which is not required to be orthogonal or normalized. Then, there exists a unique vector $|y\rangle$ satisfying the linear equations $\{\langle x_i|y\rangle = y_i\}_i$ for any $\{y_i\}_i$. Concretely, let $\{|\tilde{x}_i\rangle\}_i$ be the dual basis for $\{|x_i\rangle\}_i$, i.e., $\langle x_i|\tilde{x}_j\rangle = \delta_{ij}$, then $|y\rangle = \sum_i y_i|\tilde{x}_i\rangle$.*

First, we define $\mathcal{S}$ to be the space generated by the linearly independent operators

$$X_i = \mathcal{P}\{V^{\otimes i} \otimes \mathbb{1}^{\otimes(n-i)}\} \quad \forall\, i = 0, 1, \ldots, n, \tag{6}$$

and the inner product to be the Hilbert-Schmidt inner product, e.g.,

$$\langle X_i, X_j\rangle = \mathrm{Tr}(X_i^\dagger X_j) = \mathrm{Tr}(X_i X_j). \tag{7}$$

Then, $\Phi_{AB} \in \mathcal{S}$ by Supplementary Equation (1).

Second, we show that if Φ_{AB} exists, then it is unique. By slightly modifying the derivation of Supplementary Equation (5), it is easy to see that the normalization constraint and the marginal constraints for AME(n,d) are equivalent to

$$\mathrm{Tr}_{A_{I^c}B_{I^c}}(\Phi_{AB}) = \frac{\mathbb{1}_{d^r}}{d^r} \otimes \frac{\mathbb{1}_{d^r}}{d^r} \quad \forall\, I \in \mathcal{I}_r, \tag{8}$$

which implies that

$$\mathrm{Tr}(X_i\Phi_{AB}) = \binom{n}{i}\mathrm{Tr}\left[V^{\otimes i}\frac{\mathbb{1}_{d^i}}{d^i}\otimes\frac{\mathbb{1}_{d^i}}{d^i}\right] = \frac{\binom{n}{i}}{d^i} \quad \forall\, i = 0,1,\ldots,r. \tag{9}$$

The symmetric subspace constraint $V_{AB}\Phi_{AB} = V^{\otimes n}\Phi_{AB} = \Phi_{AB}$ and the relation $X_iV_{AB} = X_iV^{\otimes n} = X_{n-i}$ imply that

$$\mathrm{Tr}(X_i\Phi_{AB}) = \mathrm{Tr}(X_iV_{AB}\Phi_{AB}) = \mathrm{Tr}(X_{n-i}\Phi_{AB}) \quad \forall\, i = 0,1,\ldots,n. \tag{10}$$

Thus, we get

$$\langle X_i,\Phi_{AB}\rangle = \mathrm{Tr}(X_i\Phi_{AB}) = \frac{\binom{n}{i}}{\min\{d^i,d^{n-i}\}} \quad \forall\, i = 0,1,\ldots,n. \tag{11}$$

which implies the uniqueness by Lemma 1. Furthermore, in this case, we can easily write down the dual basis $\{\widetilde{X}_i\}_{i=0}^n$ for $\{X_i\}_{i=0}^n$,

$$\widetilde{X}_i = \frac{1}{\binom{n}{i}(d^2-1)^n}\mathcal{P}\left\{(\mathbb{1}-\frac{1}{d}V)^{\otimes i}\otimes(V-\frac{1}{d}\mathbb{1})^{\otimes(n-i)}\right\} \quad \forall\, i = 0,1,\ldots,n. \tag{12}$$

It is straightforward to check that $\mathrm{Tr}(\widetilde{X}_iX_j) = \delta_{ij}$. Thus, we can get an explicit form of Φ_{AB} from $x_i = \mathrm{Tr}(\widetilde{X}_i\Phi_{AB})$,

$$\begin{aligned} x_i =&\frac{1}{(d^2-1)^n}\mathrm{Tr}\left[(\mathbb{1}-\frac{1}{d}V)^{\otimes i}\otimes(V-\frac{1}{d}\mathbb{1})^{\otimes(n-i)}\Phi_{AB}\right] \\ =&\frac{1}{(d^2-1)^n}\sum_{l=0}^{n}\sum_{k=0}^{l}\frac{(-1)^{i+l}}{d^{i+l-2k}}\binom{i}{k}\binom{n-i}{l-k}\mathrm{Tr}\left[V^{\otimes(n-l)}\otimes\mathbb{1}^{\otimes l}\Phi_{AB}\right] \\ =&\frac{(-1)^i}{(d^2-1)^n}\sum_{l=0}^{n}\sum_{k=0}^{l}\frac{(-1)^l\binom{i}{k}\binom{n-i}{l-k}}{\min\{d^{i+2l-2k},d^{n+i-2k}\}}, \end{aligned} \tag{13}$$

where we have used the relation

$$\mathrm{Tr}\left[V^{\otimes(n-l)}\otimes\mathbb{1}^{\otimes l}\Phi_{AB}\right] = \frac{1}{\min\{d^l,d^{n-l}\}}, \tag{14}$$

whose proof is similar to Supplementary Equation (11).

Finally, we show the existence of Φ_{AB}, i.e., Φ_{AB} determined by Supplementary Equation (11) is compatible with the constraints in Supplementary Equations (2, 4, 5). To this end, we show that Supplementary Equation (11) implies that $V_{AB}\Phi_{AB} = \Phi_{AB}$ and Supplementary Equation (8). As $\mathrm{Tr}(X_i\Phi_{AB}) = \mathrm{Tr}(X_{n-i}\Phi_{AB})$ by Supplementary Equation (11) and $X_iV_{AB} = X_iV^{\otimes n} = X_{n-i}$, it holds that

$$\mathrm{Tr}(X_i\Phi_{AB}) = \mathrm{Tr}(X_iV_{AB}\Phi_{AB}) \quad \forall\, i = 0,1,\ldots,n. \tag{15}$$

From the uniqueness statement in Lemma 1, it follows that $V_{AB}\Phi_{AB} = \Phi_{AB}$. To prove Supplementary Equation (8), we define $\mathcal{R}$ to be the space generated by the linearly independent operators

$$R_i = \mathcal{P}\{V^{\otimes i}\otimes\mathbb{1}^{\otimes(r-i)}\} \quad \forall\, i = 0,1,\ldots,r. \tag{16}$$

Supplementary Equation (11) and the permutation symmetry of $\Phi_{AB}\in\mathcal{S}$ imply that

$$\mathrm{Tr}\left[V^{\otimes i}\otimes\mathbb{1}^{\otimes(n-i)}\Phi_{AB}\right] = \frac{1}{d^i} \quad \forall\, i = 0,1,\ldots,r. \tag{17}$$

Thus,

$$\mathrm{Tr}[R_i\,\mathrm{Tr}_{A_{I^c}B_{I^c}}(\Phi_{AB})] = \binom{r}{i}\mathrm{Tr}\left[V^{\otimes i}\otimes\mathbb{1}^{\otimes(n-i)}\Phi_{AB}\right] = \frac{\binom{r}{i}}{d^i}, \quad \forall\, i = 0,1,\ldots,r \quad \forall\, I\in\mathcal{I}_r, \tag{18}$$

Furthermore, one can easily check that

$$\mathrm{Tr}\left[R_i\frac{\mathbb{1}_{d^r}}{d^r}\otimes\frac{\mathbb{1}_{d^r}}{d^r}\right] = \frac{\binom{r}{i}}{d^i} \quad \forall\, i = 0,1,\ldots,r. \tag{19}$$

Then, applying the uniqueness statement in Lemma 1 to $\mathcal{R}$ implies Supplementary Equation (8). Hence, we proved the compatibility of Φ_{AB} with Supplementary Equations (2, 4, 5).

Supplementary Note 2: Positivity and PPT conditions for AME state

To get a closed form of the positivity and PPT conditions for AME states, we will use the following relations

$$\begin{aligned}\mathrm{Tr}\left(V^{\otimes l}\otimes \mathbb{1}^{\otimes(n-l)}\Phi_{AB}\right) &= \frac{1}{\min\{d^l,d^{n-l}\}},\\ \mathrm{Tr}\left(|\phi^+\rangle\langle\phi^+|^{\otimes l}\otimes \mathbb{1}^{\otimes(n-l)}\Phi_{AB}^{T_B}\right) &= \frac{1}{\min\{d^{2l},d^{n}\}},\end{aligned} \tag{20}$$

where the proof of the first relation is similar to Supplementary Equations (11, 17) and the second relation follows from the observation that $\mathrm{Tr}(W\Phi_{AB}^{T_B}) = \mathrm{Tr}(W^{T_B}\Phi_{AB})$. From Equation (37) it follows that the positivity condition is equivalent to $\mathrm{Tr}(P_+^{\otimes(n-i)}\otimes P_-^{\otimes i}\Phi_{AB}) \geq 0$. This gives

$$\begin{aligned}&\mathrm{Tr}\left[(\mathbb{1}+V)^{\otimes(n-i)}\otimes(\mathbb{1}-V)^{\otimes i}\Phi_{AB}\right]\\ &=\mathrm{Tr}\left[\sum_{l=0}^{n}\sum_{k=0}^{l}(-1)^k\binom{i}{k}\binom{n-i}{l-k}V^{\otimes l}\otimes\mathbb{1}^{\otimes(n-l)}\Phi_{AB}\right]\\ &=\sum_{l=0}^{n}\sum_{k=0}^{l}\frac{(-1)^k\binom{i}{k}\binom{n-i}{l-k}}{\min\{d^l,d^{n-l}\}}\geq 0 \quad \forall\, i=0,1,\ldots,n.\end{aligned} \tag{21}$$

Similarly due to Equation (38), the PPT condition is equivalent to

$$\begin{aligned}&\mathrm{Tr}\left[|\phi^+\rangle\langle\phi^+|^{\otimes(n-i)}\otimes(\mathbb{1}-|\phi^+\rangle\langle\phi^+|)^{\otimes i}\Phi_{AB}^{T_B}\right]\\ &=\mathrm{Tr}\left[\sum_{k=0}^{i}(-1)^k\binom{i}{k}|\phi^+\rangle\langle\phi^+|^{\otimes(n+k-i)}\otimes\mathbb{1}^{\otimes(i-k)}\Phi_{AB}^{T_B}\right]\\ &=\sum_{k=0}^{i}\frac{(-1)^k\binom{i}{k}}{\min\{d^{2(n+k-i)},d^n\}}\geq 0 \quad \forall\, i=0,1,\ldots,n.\end{aligned} \tag{22}$$

By noticing that

$$\mathrm{Tr}[(\mathbb{1}+V)^{\otimes(n-i)}\otimes(\mathbb{1}-V)^{\otimes i}] = d^n(d+1)^{n-i}(d-1)^i \tag{23}$$

$$\mathrm{Tr}[|\phi^+\rangle\langle\phi^+|^{\otimes(n-i)}\otimes(\mathbb{1}-|\phi^+\rangle\langle\phi^+|)^{\otimes i}] = (d^2-1)^i, \tag{24}$$

we obtain an explicit expressions for p_i and q_i

$$p_i = \frac{1}{d^n(d+1)^{n-i}(d-1)^i}\sum_{l=0}^{n}\sum_{k=0}^{l}\frac{(-1)^k\binom{i}{k}\binom{n-i}{l-k}}{\min\{d^l,d^{n-l}\}}, \tag{25}$$

$$q_i = \frac{1}{(d^2-1)^i}\sum_{k=0}^{i}\frac{(-1)^k\binom{i}{k}}{\min\{d^{2(n+k-i)},d^n\}}. \tag{26}$$

For example, for the existence of the 4-qubit AME state, the eigenvalues of the matrix Φ_{AB} are

$$(p_0,p_1,p_2,p_3,p_4) = \left(\frac{5}{864},0,\frac{1}{96},0,-\frac{1}{32}\right). \tag{27}$$

The last negative eigenvalue implies that no AME$(4,2)$ state exists.

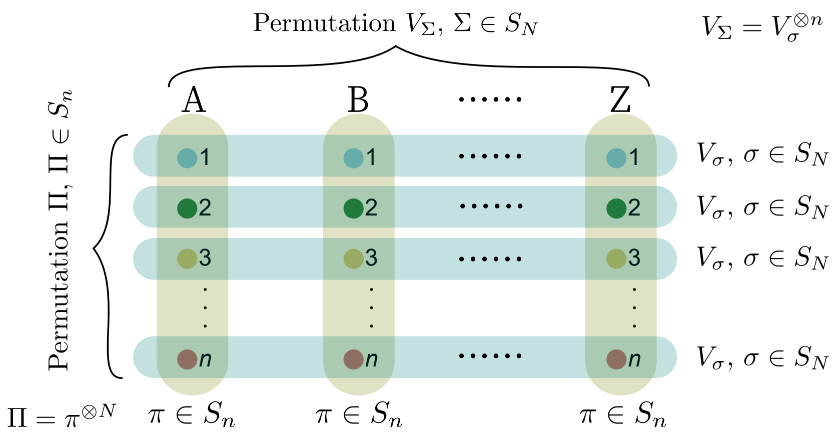

Supplementary Fig. 1. If the marginal problem has a solution $|\varphi\rangle$, then there are multi-party extensions $\Phi_{AB\cdots Z}$ for any number of copies, obeying some semidefinite constraints.

Supplementary Note 3: Multi-party extension: primal problem

We are going to analyze and simplify the hierarchy of SDPs stated in Theorem 2 for the case of the existence of AME states,

$$\begin{aligned} \text{find} \quad & \Phi_{AB\cdots Z} \\ \text{s.t.} \quad & P_N^+ \Phi_{AB\cdots Z} P_N^+ = \Phi_{AB\cdots Z}, \\ & \Phi_{AB\cdots Z} \geq 0, \ \operatorname{Tr}(\Phi_{AB\cdots Z}) = 1, \\ & \operatorname{Tr}_{A_{I^c}}(\Phi_{AB\cdots Z}) = \frac{\mathbb{1}_{d^r}}{d^r} \otimes \operatorname{Tr}(\Phi_{B\cdots Z}) \quad \forall\, I \in \mathcal{I}_r. \end{aligned} \tag{28}$$

Similar to the two-party case, we can view the N-party state $\Phi_{AB\cdots Z}$ as $\Phi_{12\ldots n}$, where i labels the subsystems $A_i B_i \cdots Z_i$. The permutations on $A_i B_i \cdots Z_i$ are denoted with subscripts $ab\cdots z$. For example, V_{AB} and V_{ABC} can be written as $V_{ab}^{\otimes n}$ and $V_{abc}^{\otimes n}$, respectively, where V_{ab} are the permutations $A_i \leftrightarrow B_i$ and V_{abc} are the permutations $A_i \to B_i \to C_i \to A_i$. Generally, we use σ and Σ to denote the permutations on $ab\cdots z$ and $AB\cdots Z$, respectively, and in addition $V_\Sigma = V_\sigma^{\otimes n}$.

Again, as the set of AME(n,d) is invariant under local unitaries and permutations on the n particles, we can assume that $\Phi_{AB\cdots Z}$ is symmetric under the following operations,

$$[U_1 \otimes \cdots \otimes U_n]^{\otimes N} \quad \forall\, U_i \in SU(d), \tag{29}$$

$$\pi^{\otimes N} \quad \forall\, \pi \in S_n. \tag{30}$$

Note that $\pi \in S_n$ denotes a permutation on $12\cdots n$ (vertical permutation in Supplementary Fig. 1), while $\sigma \in S_N$ in the previous paragraph denotes a permutation on $ab\cdots z$ (horizontal permutation in Supplementary Fig. 1). According to Schur-Weyl duality [2], any operator Φ such that $[\Phi, U^{\otimes N}] = 0$ must have the form

$$\Phi = \sum_\sigma x_\sigma V_\sigma. \tag{31}$$

Thus, the $[U_1 \otimes \cdots \otimes U_n]^{\otimes N}$ symmetry implies that

$$\Phi_{AB\cdots Z} = \sum_{\sigma_1\sigma_2\cdots\sigma_n} x_{\sigma_1\sigma_2\cdots\sigma_n} V_{\sigma_1} \otimes V_{\sigma_2} \otimes \cdots \otimes V_{\sigma_n}. \tag{32}$$

The number of parameters can be further reduced by taking advantage of the vertical permutation symmetry $\{\Pi = \pi^{\otimes N} \mid \pi \in S_n\}$, i.e.,

$$x_{\sigma_1\sigma_2\cdots\sigma_n} = x_{\sigma'_1\sigma'_2\cdots\sigma'_n} \tag{33}$$

when $\{\sigma_1, \sigma_2, \cdots, \sigma_n\}$ and $\{\sigma_1', \sigma_2', \cdots, \sigma_n'\}$ are the same multiset (set that allows repeated elements).

We are now ready to express the constraints in Supplementary Equation (28) in terms of the variables $x_{\sigma_1\sigma_2\cdots\sigma_n}$ in Supplementary Equation (32). Naively plugging Supplementary Equation (32) into Supplementary Equation (28) results in relations between large matrices; however the symmetry of the problem allows one to also simplify these constraints.

Notice that the partial trace operation can also be expressed under the basis $\{V_\sigma \mid \sigma \in S_N\}$. For example,

$$\mathrm{Tr}_c(\mathbb{1}) \otimes \mathbb{1}_c = d\mathbb{1}, \quad \mathrm{Tr}_c(V_{ab}) \otimes \mathbb{1}_c = dV_{ab}, \quad \mathrm{Tr}_c(V_{ac}) \otimes \mathbb{1}_c = \mathbb{1}, \tag{34}$$

$$\mathrm{Tr}_c(V_{bc}) \otimes \mathbb{1}_c = \mathbb{1}, \quad \mathrm{Tr}_c(V_{abc}) \otimes \mathbb{1}_c = V_{ab}, \quad \mathrm{Tr}_c(V_{cba}) \otimes \mathbb{1}_c = V_{ab}, \tag{35}$$

where all V_σ are operators on abc and we perform $\otimes\mathbb{1}_c$ to ensure that the operator stays within the original space. Similarly, we can implement the trace operation. In this way, the equality constraints regarding the marginals in Supplementary Equation (28) can be written in terms of the basis operators $V_{\sigma_1} \otimes V_{\sigma_2} \otimes \cdots \otimes V_{\sigma_n}$ without referring to explicit matrix elements. Also, the symmetric projection P_N^+ takes the form

$$P_N^+ = \frac{1}{N!} \sum_{\sigma \in S_N} V_\sigma^{\otimes n}. \tag{36}$$

Therefore the equality $P_N^+ \Phi_{AB\ldots Z} P_N^+ = \Phi_{AB\ldots Z}$ can also be expressed in terms of basis operators $V_{\sigma_1} \otimes V_{\sigma_2} \otimes \cdots \otimes V_{\sigma_n}$.

Let us now consider the positivity constraint $\Phi_{AB\ldots Z} \geq 0$. Here, the crucial observation is that $\Phi_{AB\ldots Z}$ is simply a linear combination of the basic matrices $V_{\sigma_1} \otimes V_{\sigma_2} \otimes \cdots \otimes V_{\sigma_n}$. The matrices V_{σ_i} in fact form a so-called (unitary linear) representation of the group S_N [2]. By the general theory of linear representations of groups, there is an orthogonal basis such that all of these matrices are block-diagonalized. Moreover, the possible blocks that appear in the block-diagonal form of these matrices are also completely specified by the group, known as the unitary irreducible representations of the group. In this way, the positivity constraint on $\Phi_{AB\ldots Z} \geq 0$ is reduced to the positivity of each of the different irreducible blocks.

For the symmetric group S_N, the irreducible representations are conveniently labeled by the partitions of N. A partition λ of length $k = |\lambda|$ is a tuple of positive integer numbers $\lambda = (N_1, N_2, \ldots, N_k)$ such that $N_1 \geq N_2 \geq \cdots \geq N_k$ and $N_1 + N_2 + \cdots + N_k = N$. We denote the set of all partitions by Λ_N. For each partition λ, there is an associated unitary irreducible representation M_λ, that is, the set of unitary matrices $M_\lambda(\sigma)$ for $\sigma \in S_N$. Concretely, by choosing a suitable orthonormal basis (independent of σ), all V_σ can be written as

$$V_\sigma = \bigoplus_\lambda M_\lambda(\sigma) \otimes \mathbb{1}_{d_\lambda} \tag{37}$$

where $M_\lambda(\sigma)$ correspond to the unitary irreducible representations and d_λ are the corresponding multiplicities. The matrix elements of $M_\lambda(\sigma)$ can also be constructed explicitly by taking advantage of the Young tableaux [3]. For practical purposes, these matrices can be called from an appropriate computer algebra system such as GAP [4]. For the representation V_σ, it is also known that $M_\lambda(\sigma)$ is present ($d_\lambda \neq 0$) in the block-diagonal form of V_σ if and only if the length of λ is smaller than the local dimension $|\lambda| \leq d$ [2]. We thus have the following observation.

Observation 2. *For $\Phi_{AB\ldots Z}$ in Supplementary Equation (32), $\Phi_{AB\ldots Z} \geq 0$ if and only if*

$$\sum_{\sigma_1\sigma_2\cdots\sigma_n} x_{\sigma_1\sigma_2\cdots\sigma_n} M_{\lambda_1}(\sigma_1) \otimes M_{\lambda_2}(\sigma_2) \otimes \cdots \otimes M_{\lambda_n}(\sigma_n) \geq 0, \tag{38}$$

for all $(\lambda_1, \lambda_2, \ldots, \lambda_n) \in \Lambda_N^n$ such that $|\lambda_i| \leq d$. In addition, as the state $\Phi_{AB\ldots Z}$ is also permutation-invariant under $\Pi \in S_n$, we can restrict to the cases where $\lambda_1 \geq \lambda_2 \geq \cdots \geq \lambda_n$ with any predefined order for the partitions.

There is yet another way to parameterize the optimization problem, which additionally incorporates the constraint $P_N^+ \Phi_{AB\ldots Z} P_N^+ = \Phi_{AB\ldots Z}$ more directly.

Let us recall from the above that $\Phi_{AB\ldots Z}$ as well as P_N^+ are linear combinations of operators of the form $V_{\sigma_1} \otimes V_{\sigma_2} \otimes \cdots \otimes V_{\sigma_n}$. Thus, by choosing a suitable basis such that V_{σ_i} are all block-diagonal, both $\Phi_{AB\ldots Z}$ and P_N^+ are also block-diagonal. The possible blocks of V_σ are labeled by partitions of the form $\lambda = (N_1, N_2, \ldots, N_k)$ with $k = |\lambda| \leq d$. Correspondingly, the possible blocks of $V_{\sigma_1} \otimes V_{\sigma_2} \otimes \cdots \otimes V_{\sigma_n}$ are labeled by a tuple of partitions $\boldsymbol{\lambda} = (\lambda_1, \lambda_2, \ldots, \lambda_n)$ with $|\lambda_i| \leq d$. Each of such blocks may appear multiple times, but because of Supplementary Equation (37), this simply results in exactly the same blocks in $\Phi_{AB\ldots Z}$ as well as P_N^+. Therefore, considering just one time of appearance of each block is sufficient. Moreover, because of the symmetry of coefficients in the linear combination under vertical permutations as in Supplementary Equation (33), only a single representative of the

tuples of partitions that are different by a vertical permutation needs to be considered. Hence, we are left with analyzing the constraint $P_N^+ \Phi_{AB\cdots Z} P_N^+ = \Phi_{AB\cdots Z}$ within the blocks corresponding to $\boldsymbol{\lambda} = (\lambda_1, \lambda_2, \ldots, \lambda_n)$.

More specifically, let $\mathcal{H}_{\lambda_i}$ denote the subspace corresponding to the blocks λ_i of the operators V_{σ_i}. Then the subspace corresponding to the block $\boldsymbol{\lambda} = (\lambda_1, \lambda_2, \ldots, \lambda_n)$ of $V_{\sigma_1} \otimes V_{\sigma_2} \otimes \cdots \otimes V_{\sigma_n}$ is given by

$$\mathcal{H}_{\boldsymbol{\lambda}} = \mathcal{H}_{\lambda_1} \otimes \mathcal{H}_{\lambda_2} \otimes \cdots \otimes \mathcal{H}_{\lambda_n}. \tag{39}$$

In this subspace, the symmetric projection P_N^+ reads

$$(P_N^+)^{\boldsymbol{\lambda}} = \frac{1}{N!} \sum_{\sigma \in S_N} M_{\lambda_1}(\sigma) \otimes M_{\lambda_2}(\sigma) \cdots \otimes M_{\lambda_n}(\sigma). \tag{40}$$

The constraint $P_N^+ \Phi_{AB\cdots Z} P_N^+ = \Phi_{AB\cdots Z}$ restricted to the subspace $\mathcal{H}_{\boldsymbol{\lambda}}$ means that the corresponding block of $\Phi_{AB\cdots Z}$, denoted as $\Phi_{AB\cdots Z}^{\boldsymbol{\lambda}}$, is supported only on the symmetric subspace defined by the projection $(P_N^+)^{\boldsymbol{\lambda}}$,

$$\mathcal{K}_{\boldsymbol{\lambda}} = \text{Image}\left[(P_N^+)^{\boldsymbol{\lambda}}\right]. \tag{41}$$

Thus, if one chooses a basis $\{|\Psi_i^{\boldsymbol{\lambda}}\rangle\}_{i=1}^{k_{\boldsymbol{\lambda}}}$, where $k_{\boldsymbol{\lambda}} = \dim(\mathcal{K}_{\boldsymbol{\lambda}})$, for this subspace $\mathcal{K}_{\boldsymbol{\lambda}}$, then the corresponding block of $\Phi_{AB\cdots Z}$ is of the form

$$\Phi_{AB\cdots Z}^{\boldsymbol{\lambda}} = \sum_{i,j=1}^{k_{\boldsymbol{\lambda}}} X_{ij}^{\boldsymbol{\lambda}} |\Psi_i^{\boldsymbol{\lambda}}\rangle\langle\Psi_j^{\boldsymbol{\lambda}}|. \tag{42}$$

In this way, $\Phi_{AB\cdots Z}^{\boldsymbol{\lambda}}$ is parameterized by the matrix $X^{\boldsymbol{\lambda}}$, and its positivity reduces to the positivity of $X^{\boldsymbol{\lambda}}$.

In short, let us summarize the procedure to implement the optimization problem. First, enumerate all irreducible representations of S_N, i.e., all possible partitions λ. Then, select those partitions that have length $|\lambda|$ no longer than d. Based on that, enumerate all tuples of partitions $\boldsymbol{\lambda} = (\lambda_1, \lambda_2, \ldots, \lambda_n)$ with $|\lambda_i| \leq d$. For each of those tuples $\boldsymbol{\lambda}$, compute the symmetric projection $(P_N^+)^{\boldsymbol{\lambda}}$ by Supplementary Equation (40) and select a basis for $\mathcal{K}_{\boldsymbol{\lambda}} = \text{Image}(P_N^+)^{\boldsymbol{\lambda}}$. Finally, for each partition tuple $\boldsymbol{\lambda}$, consider the associated positive semidefinite Hermitian matrix variable $X^{\boldsymbol{\lambda}}$ and write down the constraints corresponding to the condition on the marginals in Supplementary Equation (28) to complete the SDP.

In addition, we provide some more details for the construction of the basis of $\mathcal{K}_{\boldsymbol{\lambda}}$. For readers who are familiar with the representation theory of groups, there is a simple characterization of $\mathcal{K}_{\boldsymbol{\lambda}}$ that helps carrying out the practical implementation. In the language of representation theory, $\mathcal{H}_{\lambda_i}$ is an irreducible representation of S_N, while $\mathcal{H}_{\boldsymbol{\lambda}}$ is an irreducible representation of $(S_N)^n$. This space is also a representation of S_N via the diagonal embedding into $(S_N)^n$, which maps $\sigma \in S_N$ to $(\sigma, \sigma, \ldots, \sigma) \in (S_N)^n$. As a representation of S_N, $\mathcal{H}_{\boldsymbol{\lambda}}$ contains a subrepresentation $\mathcal{K}_{\boldsymbol{\lambda}}$ on which S_N acts trivially (this is technically known as the isotropic component of the trivial representation). Methods of representation theory then allow for detailed characterization of $\mathcal{K}_{\boldsymbol{\lambda}}$. In particular, one obtains the dimension of $\mathcal{K}_{\boldsymbol{\lambda}}$ as [2]

$$k_{\boldsymbol{\lambda}} = \frac{1}{N!} \sum_{\sigma \in S_N} \prod_{i=1}^{n} \text{Tr}(M_{\lambda_i}(\sigma)). \tag{43}$$

The symmetric projection $(P_N^+)^{\boldsymbol{\lambda}}$ in Supplementary Equation (40) is in fact also known as the twirling operator: it maps a vector of $\mathcal{H}_{\boldsymbol{\lambda}}$ to its average under the action of the group S_N. A basis of this space can be found by applying the twirling operation $(P_N^+)^{\boldsymbol{\lambda}}$ to a set of $k^{\boldsymbol{\lambda}}$ random vectors in $\mathcal{H}_{\boldsymbol{\lambda}}$; if the resulted vectors are linearly independent, they form a basis of $\mathcal{K}_{\lambda}$, else one can start over with another random set of vectors. As an alternative method, Supplementary Equations (40, 41) imply that $\mathcal{K}_{\boldsymbol{\lambda}}$ is the common unit eigenspace of $M_{\lambda_1}(\sigma) \otimes M_{\lambda_2}(\sigma) \otimes \cdots \otimes M_{\lambda_n}(\sigma)$ for all $\sigma \in S_N$. As all eigenvalues of $M_{\lambda_i}(\sigma)$ are always in the unit circle, a basis of $\mathcal{K}_{\boldsymbol{\lambda}}$ can also constructed from calculating the kernel of

$$M_{\lambda_1}(\sigma_s) \otimes M_{\lambda_2}(\sigma_s) \otimes \cdots \otimes M_{\lambda_n}(\sigma_s) + M_{\lambda_1}(\sigma_c) \otimes M_{\lambda_2}(\sigma_c) \otimes \cdots \otimes M_{\lambda_n}(\sigma_c) - 2\mathbb{1}, \tag{44}$$

where $\sigma_s = (ab)$ and $\sigma_c = (ab \cdots z)$ form a set of generators of S_N.

As another technical remark, working with unitary representation requires computation with cyclotomic numbers, which is often slow. Therefore, one may adjust the procedure by implementing intermediate computations in non-unitary representations (or equivalently, working in non-orthogonal bases) where matrix elements (of the representations of symmetric groups) are all rationals.

Supplementary Note 4: Multi-party extension: dual problem and entanglement witness

Specifically for the existence problem of AME states, as Φ_{AB} is uniquely determined, one can easily verify that the following equation is a relaxed but still complete hierarchy of Theorem 2,

$$\begin{aligned} \text{find} \quad & \Phi_{ABC\cdots Z} \\ \text{s.t.} \quad & \mathrm{Tr}_{C\cdots Z}(P_N^+ \Phi_{ABC\cdots Z} P_N^+) = \Phi_{AB}, \\ & P_N^+ \Phi_{ABC\cdots Z} P_N^+ \geq 0, \end{aligned} \tag{45}$$

where Φ_{AB} is the unique quantum state given by Theorem 3. Alternatively, we can write the objective function in Supplementary Equation (45) as $\max_{\Phi_{ABC\cdots Z}}\{0\}$, such that the dual problem reads

$$\begin{aligned} \min_{W_{AB}} \quad & \mathrm{Tr}(W_{AB}\Phi_{AB}) \\ \text{s.t.} \quad & P_N^+ W_{AB} \otimes \mathbb{1}_{C\cdots Z} P_N^+ \geq 0, \end{aligned} \tag{46}$$

where W_{AB} is Hermitian. One can easily verify that strong duality holds from Slater's condition [5] with positivity considered on the symmetric subspace, which means the problem in Supplementary Equation (45) is feasible if and only if the solution of the dual problem in Supplementary Equation (46) equals zero. Thus, if $\mathrm{Tr}(W_{AB}\Phi_{AB}) < 0$, we know that Φ_{AB} is entangled and the corresponding AME state does not exist from Theorem 3. Notice that numerically determining the negativity of the dual problem in Supplementary Equation (46) is less sensitive to small numerical errors, and hence, more stable than solving the primal feasibility problem in Supplementary Equation (45). Moreover, the physical meaning of W_{AB} is also clear: a feasible point W_{AB} of Supplementary Equation (46) with a negative objective value provides an entanglement witness for Φ_{AB} in the symmetric subspace $P_2^+ = \frac{1}{2}(\mathbb{1}_{AB} + V_{AB})$. Indeed, because the set of separable states in P_2^+ is given by $\mathrm{conv}\{|\psi\rangle\langle\psi| \otimes |\psi\rangle\langle\psi|\}$, the constraint in Supplementary Equation (46) implies that

$$\langle\psi|\langle\psi|W_{AB}|\psi\rangle|\psi\rangle = \langle\psi|^{\otimes N} P_N^+ W_{AB} \otimes \mathbb{1}_{C\cdots Z} P_N^+ |\psi\rangle^{\otimes N} \geq 0. \tag{47}$$

The analysis of the symmetry and parametrization of the dual problem Supplementary Equation (46) is similar to that for the primal problem as discussed in Supplementary Note 3; in fact, it is more straightforward for the dual problem. For $g \in G$ defined in Supplementary Equations (31, 32), we have

$$g\Phi_{AB}g^\dagger = \Phi_{AB}, \quad gP_N^+ g^\dagger = P_N^+. \tag{48}$$

In addition, we know that Φ_{AB} and P_N^+ are also in the symmetric subspace P_2^+, i.e.,

$$P_2^+ \Phi_{AB} P_2^+ = \Phi_{AB}, \quad \left(P_2^+ \otimes \mathbb{1}_{C\cdots Z}\right) P_N^+ \left(P_2^+ \otimes \mathbb{1}_{C\cdots Z}\right) = P_N^+. \tag{49}$$

Thus, we can assume that W_{AB} is invariant under G and constrained to P_2^+, i.e.,

$$gW_{AB}g^\dagger = W_{AB} \quad \forall\, g \in G, \quad P_2^+ W_{AB} P_2^+ = W_{AB}. \tag{50}$$

Similar to the analysis of Equation (35), one can easily see that $gW_{AB}g^\dagger = W_{AB}$ for all $g \in G$ implying that

$$W_{AB} = \sum_{l=0}^{n} w_l \mathcal{P}\{V^{\otimes l} \otimes \mathbb{1}^{\otimes(n-l)}\}, \tag{51}$$

where again $\mathcal{P}$ denotes the sum over all permutations of the tensor product under its argument. Furthermore, $P_+ W_{AB} P_+ = W_{AB}$ implies that

$$w_l = w_{n-l} \quad \forall\, l = 0, 1, \ldots, n-r-1. \tag{52}$$

Hence, the objective function $\mathrm{Tr}(W_{AB}\Phi_{AB})$ can be expressed as

$$\mathrm{Tr}(W_{AB}\Phi_{AB}) = \sum_{l=0}^{n} a_l w_l, \tag{53}$$

where

$$a_l = \mathrm{Tr}(\mathcal{P}\{V^{\otimes l} \otimes \mathbb{1}^{\otimes(n-l)}\}\Phi_{AB}) = \frac{\binom{n}{l}}{\min\{d^l, d^{n-l}\}}, \tag{54}$$

from Supplementary Equation (11).

To get some intuition about the variables w_l, let us consider the problem of the existence of AME(4,6). Here $n = 4$ and hence, there are five variables w_l in Supplementary Equation (51). Moreover, Supplementary Equation (52) implies that only three of those variables are independent. Furthermore, one can notice that the dual problem in Supplementary Equation (46) is homogeneous, that is, the objective function is linear and the constraints are invariant under rescaling $W_{AB} \to tW_{AB}$ with $t > 0$. This allows one to impose that $w_0 = 0$ or $w_0 = \pm 1$, and one is then left with two independent variables.

The constraint $P_N^+ W_{AB} \otimes \mathbb{1}_{C\cdots Z} P_N^+ \geq 0$ can be expressed in terms of the variables w_l in similarity to Supplementary Note 3. Let us summarize the arguments once more for completeness. The fact that $W_{AB} \otimes \mathbb{1}_{C\cdots Z}$ and P_N^+ are both of linear combinations of $V_{\sigma_1} \otimes V_{\sigma_2} \otimes \cdots \otimes V_{\sigma_n}$ implies that they are block-diagonal when one chooses a basis such that the V_{σ_i} are block-diagonal. Let $\mathcal{H}_{\lambda_i}$ denote the subspace corresponding to the block of V_{σ_i} labeled by partition λ_i with $|\lambda_i| \leq d$. Then $\mathcal{H}_{\boldsymbol{\lambda}} = \mathcal{H}_{\lambda_1} \otimes \mathcal{H}_{\lambda_2} \otimes \mathcal{H}_{\lambda_n}$ denotes the subspace corresponding to a block of $V_{\sigma_1} \otimes V_{\sigma_2} \otimes \cdots \otimes V_{\sigma_n}$ labeled by a tuple of partitions $\boldsymbol{\lambda} = (\lambda_1, \lambda_2, \ldots, \lambda_n)$. Moreover, within this subspace, $(P_N^+)^{\boldsymbol{\lambda}}$ is a projection onto the symmetric subspace, which is typically low-rank. Let $\mathcal{K}_{\boldsymbol{\lambda}}$ denote the image of $(P_N^+)^{\boldsymbol{\lambda}}$ and $\{|\Psi_i^{\boldsymbol{\lambda}}\rangle\}_{i=1}^{k_{\boldsymbol{\lambda}}}$ denote a basis of $\mathcal{K}_{\boldsymbol{\lambda}}$. One defines the matrix $Y^{\boldsymbol{\lambda}}$ as

$$Y_{ij}^{\boldsymbol{\lambda}} = \langle \Psi_i^{\boldsymbol{\lambda}} | P_N^+ W_{AB} \otimes \mathbb{1}_{C\cdots Z} P_N^+ | \Psi_j^{\boldsymbol{\lambda}} \rangle. \tag{55}$$

Notice that in computing these matrix elements, we only need the blocks of P_N^+ and $W_{AB} \otimes \mathbb{1}_{C\cdots Z}$ corresponding to partitions $\boldsymbol{\lambda}$. Then, $P_N^+ W_{AB} \otimes \mathbb{1}_{C\cdots Z} P_N^+ \geq 0$ is equivalent to $Y^{\boldsymbol{\lambda}} \geq 0$ for all tuples of partitions $\boldsymbol{\lambda}$ with $|\lambda_i| \leq d$. Moreover, since the problem is symmetric under vertical permutations, tuples of partitions $\boldsymbol{\lambda}$ that are different by a vertical permutation are considered just once.

As a final remark, we can consider the relaxations of the constraints in Supplementary Equation (46). If the optimal value of a relaxed problem is non-negative, we conclude that the optimal value of Supplementary Equation (46) is also non-negative. In particular, ignoring some tuples of partitions $\boldsymbol{\lambda}$ in the constraints $Y^{\boldsymbol{\lambda}} \geq 0$ corresponds to a relaxation of Supplementary Equation (46). For example, one can consider only $\boldsymbol{\lambda}$ such that $(P_N^+)^{\boldsymbol{\lambda}}$ is rank-1 and obtain a linear program relaxation of Supplementary Equation (46).

Supplementary Note 5: Failed approaches to the AME problem

In this section, we discuss the approaches that we applied to investigate the separability of states which encode the existence of AME states. For the interesting case of AME$(4,6)$, however, none of them delivers a solution to the problem.

A. The state for the AME$(4,6)$ problem

Let us start by recalling the state presented already in Corollary 4. The state acts on a $6^4 \times 6^4$ system, where Alice and Bob each own four six-dimensional systems. The state is given by

$$\Phi_{AB} = \frac{1}{2 \cdot 6^4} \left(\frac{P_+^{\otimes 4}}{343} + \frac{\mathcal{P}\{P_+^{\otimes 2} \otimes P_-^{\otimes 2}\}}{315} + \frac{P_-^{\otimes 4}}{375} \right), \tag{56}$$

where $P_\pm$ are the projectors onto the (anti-)symmetric subspace of the 6×6 systems. Here, the tensor product denotes the tensor product between the four 6×6 systems and $\mathcal{P}\{\cdot\}$ denotes a sum over all permutations of the four copies that give distinct terms; in this case, there are six different terms. Note that the state Φ_{AB} acts on the symmetric subspace only.

It is also useful to consider the partial transposition of this state. Let $|\phi^+\rangle = (\sum_{k=0}^{5} |kk\rangle)/\sqrt{6}$ be the maximally entangled state of two six-dimensional systems and define $P_\perp = \mathbb{1} - |\phi^+\rangle\langle\phi^+|$ as the projector onto the corresponding

orthogonal subspace. Then, we have

$$\Phi_{AB}^{T_B} = \frac{1}{6^4}\left(|\phi^+\rangle\langle\phi^+|^{\otimes 4} + \frac{\mathcal{P}\{|\phi^+\rangle\langle\phi^+|\otimes P_\perp^{\otimes 3}\}}{35^2} + \frac{33 P_\perp^{\otimes 4}}{35^3}\right). \tag{57}$$

This time, the sum over all permutations contains four different terms. Clearly, the separability of Φ_{AB} is equivalent to the separability of $\Phi_{AB}^{T_B}$. To test whether or not these states are entangled the following approaches came to our mind:

- The state $\Phi_{AB}^{T_B}$ has a similarity to the states discussed in Ref. [6]. There, a family of bound entangled states with high Schmidt rank has been constructed. To do so, one considers a bipartite system, where Alice's as well as Bob's system can be further split up into two subsystems, A_1 and A_2 as well as B_1 and B_2, respectively. Then, one investigates unnormalized states of the form

$$Z = X_{A_1B_1} \otimes (P_\perp)_{A_2B_2} + Y_{A_1B_1} \otimes |\phi^+\rangle\langle\phi^+|_{A_2B_2}. \tag{58}$$

  Under weak conditions on $X_{A_1B_1}$ and $Y_{A_1B_1}$ one can show that Z is a bipartite entangled state with a positive partial transpose. For instance, one may choose $X_{A_1B_1} = (P_\perp)_{A_1B_1}$ and $Y_{A_1B_1} = (d_1-1)(d_2+1)|\phi^+\rangle\langle\phi^+|_{A_1B_1}$. Here, d_1 is the dimension of A_1 and B_1 and d_2 the dimension of A_2 and B_2. For the argument of Ref. [6] it is crucial that these dimensions are different, typically one takes $d_2 \gg d_1$.

  The entanglement proof for the states in Ref. [6] goes as follows: The map

$$\Lambda(\cdot) = \mathbb{1}\,\mathrm{Tr}(\cdot) - \frac{1}{k}\mathrm{id}(\cdot), \tag{59}$$

  is k-positive, where $\mathrm{id}(\cdot)$ denotes the identity map. That is, the output of $\mathrm{id}\otimes\Lambda$ is always positive on states with Schmidt rank k. A non-positive output by applying this map to the A_2B_2 part of states of the form in Supplementary Equation (58), i.e., applying $\mathrm{id}_{A_1B_1A_2}\otimes\Lambda_{B_2}$, would indicate that the state has a very high Schmidt rank in the systems A_2B_2. The (low-dimensional) systems A_1B_1 cannot significantly change the Schmidt rank, so the total state must be entangled. This idea can also be formalized by writing down explicit entanglement witnesses [6].

  For the state $\Phi_{AB}^{T_B}$ one can apply similar tricks. For instance, one can split the four subsystems of Alice and Bob in a one-vs-three partition to achieve $d_2 \gg d_1$. In this particular case, however, the state is not detected as entangled, the expectation value of the witness from Ref. [6] vanishes. One may also consider further refined splits, as any six-dimensional system can be seen as a (2×3)-system. For example, one can split the system such that $d_1 = 2^4 = 16$ and $d_2 = 3^4 = 81$. Still, we found no proof of entanglement for $\Phi_{AB}^{T_B}$, however, the expectation value for several of the resulting witnesses vanishes.

- Similar states as in Ref. [6] were also considered before in Ref. [7]. There, entanglement witnesses of the form

$$W = |\psi_1\rangle\langle\psi_1|_{A_1B_1}\otimes\mathbb{1}_{A_2B_2} - (1+\varepsilon)|\psi_1\rangle\langle\psi_1|_{A_1B_1}\otimes|\psi_2\rangle\langle\psi_2|_{A_2B_2} \tag{60}$$

  have been investigated. For the purpose of Ref. [7], it was only relevant that for some $\varepsilon > 0$ this operator is indeed positive on all separable states, and it was shown that this holds for nearly arbitrary $|\psi_1\rangle$ and $|\psi_2\rangle$.

  For our purposes, we need to calculate the maximal ε explicitly. If we assume that $|\psi_1\rangle$ and $|\psi_2\rangle$ are maximally entangled states in different dimensions, this can be done as follows: First, we know that $W_k = k/d_2 - |\psi_2\rangle\langle\psi_2|$ is a Schmidt rank-k witness. Second, if we consider a product state $|\eta\rangle = |\alpha\rangle_{A_1A_2}\otimes|\beta\rangle_{B_1B_2}$, the unnormalized pure state

$$|\zeta\rangle\langle\zeta|_{A_2B_2} = \mathrm{Tr}_{A_1B_1}\left[|\eta\rangle\langle\eta|_{A_1A_2B_1B_2}|\psi_1\rangle\langle\psi_1|_{A_1B_1}\right], \tag{61}$$

  has at most Schmidt rank d_1. Combining these observations, we find that W in Supplementary Equation (60) is an entanglement witness if

$$\varepsilon \leq \frac{d_2}{d_1} - 1. \tag{62}$$

  For instance, taking the state $\Phi_{AB}^{T_B}$ as well as $d_1 = 2$ and $d_2 = 6^3\times 3$, one obtains $\varepsilon = 323$. Still, we find $\mathrm{Tr}(W\Phi_{AB}^{T_B}) = 0$ and no entanglement is detected.

- As described in Supplementary Note 3, we also tested whether or not there exists a symmetric extension for the state Φ_{AB} making use of the symmetries to reduce the number of parameters substantially. However, for large extensions, computing the bases for $\mathcal{K}_{\boldsymbol{\lambda}}$ in Supplementary Equation (41) as well as rephrasing the constraints in terms of the variables in Supplementary Equation (42) takes a considerable amount of time. Moreover, precision issues pose a major challenge due to coefficients being of different order of magnitude.

  One possible relaxation that simplifies the computation is to consider the second last constraint in the SDP in Supplementary Equations (28) only for some marginal of the extension. The largest extension we computed reliably is $N = 5$ while restricting the second last constraint to $\Phi_{ABC} = \mathrm{Tr}_{DE}(\Phi_{ABCDE})$. Furthermore, we computed a PPT-extension for $N = 3$ utilizing the basis from Ref. [8]. Both of these extensions exist up to numerical precision.

- We implemented the dual problem in Supplementary Equation (46) exploiting its symmetry as discussed in Supplementary Note 4. Using the linear program relaxation of the problem by means of retaining only partitions $\boldsymbol{\lambda}$ such that the symmetric projection $(P_N^+)^{\boldsymbol{\lambda}}$ is rank-1 as discussed there, we can show that the optimal values are non-negative up to $N = 7$. Thus the hierarchy fails to indicate the possible entanglement of Φ_{AB} up to $N = 7$.

- A final idea could be to start with the symmetric state Φ_{AB} and use the following strategy to prove that the state is entangled: For a multiparticle symmetric state it is known that it is either fully separable or genuine multipartite entangled. This implies that if a multiparticle symmetric state is entangled for one bipartition, it must be entangled for all bipartitions. Hence, proving entanglement for one bipartition can be used to show entanglement for another bipartition, even if the state has a positive partial transpose for the latter bipartition. This trick has been exploited to find symmetric bound entangled states [9].

  For the state Φ_{AB} one would need to find an embedding in a multiparticle system, where Φ_{AB} corresponds to some bipartition. This, however, is not straightforward, as the embedding idea from Ref. [9] does not work for bipartite symmetric states with maximal rank.

B. The state for the $\mathrm{AME}(7,2)$ problem

For training purposes, it may be useful to consider a state where the separability properties are known. The following state originates from the seven-qubit AME problem, where no AME state exists [10]. It is, however, not easy to see the entanglement of the corresponding state directly, and finding a criterion might also help to decide whether or not there is an $\mathrm{AME}(4,6)$ state.

The state acts on a $2^7 \times 2^7$ system, where Alice and Bob each own seven qubits:

$$\Phi_{AB} = \frac{113}{1119744} P_+^{\otimes 7} + \frac{17}{124416} \mathcal{P}\left\{P_+^{\otimes 5} \otimes P_-^{\otimes 2}\right\} + \frac{1}{13824} \mathcal{P}\left\{P_+^{\otimes 3} \otimes P_-^{\otimes 4}\right\} + \frac{1}{1536} \mathcal{P}\left\{P_+^{\otimes 1} \otimes P_-^{\otimes 6}\right\}, \tag{63}$$

where $P_\pm$ are the projectors onto the (anti-)symmetric subspace of the 2×2 systems.

For the partial transposition, let $|\phi^+\rangle = (|00\rangle + |11\rangle)/\sqrt{2}$ be the two-qubit Bell state, and $P_\perp = \mathbb{1} - |\phi^+\rangle\langle\phi^+|$ the projector onto the corresponding orthogonal subspace. Then,

$$\begin{aligned}\Phi_{AB}^{T_B} = &\frac{1}{128} |\phi^+\rangle\langle\phi^+|^{\otimes 7} + \frac{1}{10368} \mathcal{P}\left\{|\phi^+\rangle\langle\phi^+|^{\otimes 3} \otimes P_\perp^{\otimes 4}\right\} + \frac{1}{15552} \mathcal{P}\left\{|\phi^+\rangle\langle\phi^+|^{\otimes 2} \otimes P_\perp^{\otimes 5}\right\} \\ &+ \frac{1}{23328} \mathcal{P}\left\{|\phi^+\rangle\langle\phi^+| \otimes P_\perp^{\otimes 6}\right\} + \frac{11}{139968} P_\perp^{\otimes 7}.\end{aligned} \tag{64}$$

As no $\mathrm{AME}(7,2)$ state exists, the states Φ_{AB} and $\Phi_{AB}^{T_B}$ in Supplementary Equations (63, 64) are entangled, but we are not aware of any operational entanglement criterion detecting them.

Supplementary References

[1] Lebedev, L. P., Cloud, M. J., and Eremeyev, V. A., *Tensor analysis with applications in mechanics* (World Scientific, Singapore, 2010).

[2] Fulton, W. and Harris, J., *Representation theory: a first course*, Vol. 129 (Springer-Verlag, Berlin, 1991).

[3] Boerner, H., *Representations of groups* (North-Holland, Amsterdam, 1963).
[4] GAP, *GAP – Groups, Algorithms, and Programming, Version 4.11.0*, The GAP Group (2020), https://www.gap-system.org.
[5] Boyd, S. and Vandenberghe, L., *Convex optimization* (Cambridge University Press, New York, 2004).
[6] Huber, M., Lami, L., Lancien, C., and Müller-Hermes, A., "High-dimensional entanglement in states with positive partial transposition," Phys. Rev. Lett. **121**, 200503 (2018).
[7] Piani, M. and Mora, C. E., "Class of positive-partial-transpose bound entangled states associated with almost any set of pure entangled states," Phys. Rev. A **75**, 012305 (2007).
[8] Eggeling, T. and Werner, R. F., "Separability properties of tripartite states with $u \otimes u \otimes u$ symmetry," Phys. Rev. A **63**, 042111 (2001).
[9] Tóth, G. and Gühne, O., "Entanglement and permutational symmetry," Phys. Rev. Lett. **102**, 170503 (2009).
[10] Huber, F., Gühne, O., and Siewert, J., "Absolutely maximally entangled states of seven qubits do not exist," Phys. Rev. Lett. **118**, 200502 (2017).
